# Evaluation of Conspiracy Beliefs, Vaccine Hesitancy, and Willingness to Pay towards COVID-19 Vaccines in Six Countries from Asian and African Regions: A Large Multinational Analysis

**DOI:** 10.3390/vaccines10111866

**Published:** 2022-11-04

**Authors:** Muhammad Salman, Tauqeer Hussain Mallhi, Nida Tanveer, Naureen Shehzadi, Humaira Majeed Khan, Zia Ul Mustafa, Tahir Mehmood Khan, Khalid Hussain, Malik Suliman Mohamed, Faheem Maqbool, Raja Ahsan Aftab, Muhammad Hammad Butt, Dibya Sundar Panda, Nasser Hadal Alotaibi, Amgad I. M. Khedr, Abdullah Salah Alanazi, Ahmed D. Alatawi, Abdulaziz Ibrahim Alzarea, Kishwar Sulatana, Yusra Habib Khan

**Affiliations:** 1 Institute of Pharmacy, Faculty of Pharmaceutical and Allied Health Sciences, Lahore College for Women University, Lahore 54000, Pakistan; 2 Department of Clinical Pharmacy, College of Pharmacy, Jouf University, Sakaka 72388, Al-Jouf Province, Saudi Arabia; 3 Institute of Molecular Cardiology, University of Louisville, Louisville, KY 40202, USA; 4 College of Pharmacy, University of the Punjab, Lahore 54000, Pakistan; 5 Department of Pharmacy Services, District Headquarter Hospital, Pakpattan 57400, Pakistan; 6 Institute of Pharmaceutical Science, University of Veterinary and Animal Sciences, Lahore 54000, Pakistan; 7 Department of Pharmaceutics, College of Pharmacy, Jouf University, Sakaka 72388, Al-Jouf Province, Saudi Arabia; 8 School of Pharmacy, The University of Queensland, Woolloongabba, QLD 4102, Australia; 9 School of Pharmacy, Taylor’s University, Selangor 47500, Malaysia; 10 Department of Medicinal Chemistry, Faculty of Pharmacy, Uppsala University, 75123 Uppsala, Sweden; 11 Department of Pharmacognosy, Faculty of pharmacy, Port Said University, Port Said 42526, Egypt; 12 Faculty of Pharmacy, The University of Lahore, 1 km Defense Road, Lahore 54000, Pakistan

**Keywords:** vaccine hesitancy, vaccination, COVID-19, conspiracy beliefs, willingness to pay, conspiracy theories, Africa, Asia

## Abstract

Vaccination protects people from serious illness and associated complications. Conspiracy theories and misinformation on vaccines have been rampant during the COVID-19 pandemic and are considered significant drivers of vaccine hesitancy. Since vaccine hesitancy can undermine efforts to immunize the population against COVID-19 and interferes with the vaccination rate, this study aimed to ascertain the COVID-19-vaccine-related conspiracy beliefs, vaccine hesitancy, views regarding vaccine mandates, and willingness to pay for vaccines among the general population. A web-based, cross-sectional survey was conducted (April–August 2021) among the adult population in six countries (Pakistan, Saudi Arabia, India, Malaysia, Sudan, and Egypt). Participants were recruited using an exponential, non-discriminate snowball sampling method. A validated self-completed electronic questionnaire was used for the data collection. All the participants responded to questions on various domains of the study instrument, including conspiracy beliefs, vaccine hesitancy, and willingness to pay. The responses were scored according to predefined criteria and stratified into various groups. All data were entered and analyzed using SPSS version 22. A total of 2481 responses were included in the study (Pakistan 24.1%, Saudi Arabia 19.5%, India 11.6%, Malaysia 8.1%, Sudan 19.3%, and Egypt 17.3%). There was a preponderance of participants ≤40 years old (18–25 years: 55.8%, 26–40 years: 28.5%) and females (57.1%). The average score of the COVID-19 vaccine conspiracy belief scale (C19V-CBS) was 2.30 ± 2.12 (median 2; range 0–7). Our analysis showed that 30% of the respondents were found to achieve the ideal score of zero, indicating no conspiracy belief. The mean score of the COVID-19 vaccine hesitancy scale (C19V-HS) was 25.93 ± 8.11 (range: 10–50). The majority (45.7%) had C19V-HA scores of 21–30 and nearly 28% achieved a score greater than 30, indicating a higher degree of hesitancy. There was a significant positive correlation between conspiracy beliefs and vaccine hesitancy (Spearman’s rho = 0.547, *p* < 0.001). Half of the study population were against the vaccine mandate. Respondents in favor of governmental enforcement of COVID-19 vaccines had significantly (*p* < 0.001) lower scores on the C19V-CBS and C19V-HS scale. Nearly 52% reported that they would only take vaccine if it were free, and only 24% were willing to pay for COVID-19 vaccines. A high prevalence of conspiracy beliefs and vaccine hesitancy was observed in the targeted countries. Our findings highlight the dire need for aggressive measures to counter the conspiracy beliefs and factors underlying this vaccine hesitancy.

## 1. Introduction

Since the emergence of SARS-CoV-2 in 2019, intense scientific innovation and rigorous testing by several pharmaceutical industries has resulted in the development of various vaccines against COVID-19 [1]. The mRNA-based and adenovector vaccines have demonstrated satisfactory outcomes in clinical trials [2,3,4,5,6]. Currently, vaccinating the population is the utmost priority for all healthcare authorities around the globe. Moreover, the emergence of novel variants has inclined many countries toward booster doses [7]. However, the willingness to vaccinate and confidence in the vaccines are key players in national and international vaccination campaigns. Vaccination hesitancy started to become a significant issue during the COVID-19 pandemic. It must be noted that the utility of vaccine campaigns in curbing the COVID-19 does not merely rely on the safety and efficacy of the vaccines. The confidence of the public, as well as healthcare professionals, plays a decisive role in the success of any vaccination campaign [8]. Since the vaccines have been developed in record time, various conspiracy theorists have taken the opportunity to spread misleading narratives about the COVID-19 and its vaccines among the general community [9]. It has been established that people’s beliefs in conspiracy theories about infectious diseases negatively impact their health behaviors concerning vaccination. Vaccine hesitancy due to conspiracy beliefs has become more rampant during the COVID-19 pandemic [10]. Neutralizing the conspiracy beliefs and assessing vaccine hesitancy in the general population is a direly needed measure during the ongoing pandemic.

Since the effective control of COVID-19 relies heavily on herd immunity, vaccines are being considered as the one and only measure to achieve such immunity, as waiting for the population to contract the virus for herd immunity is relatively risky, particularly when children account for a large proportion of the country’s population. However, reaching a herd immunity threshold appears to be difficult because of various challenges, such as vaccine hesitancy, the emergence of new variants, and the delayed arrival of vaccinations for children [11]. For a vaccination program to succeed, the implementers should consider the priority of understanding and contextualizing the range, breadth, and depth of circulating misinformation, conspiracy theories, and vaccine hesitancy in the community. We believe that the vaccination efforts should be a composite of ensuring the easy access and availability of the vaccines, understanding the public’s hesitancy and beliefs, and addressing them promptly through a series of interventions. In these contexts, this study aimed to ascertain the conspiracy beliefs and vaccine hesitancy among the general population of six Asian countries, including Pakistan, India, Saudi Arabia, Sudan, Egypt, and Malaysia. Since the COVID-19 vaccines’ acceptance level is moderate to low in these countries, this study provides an assessment of the recent pattern of vaccine hesitancy in these regions.

## 2. Materials and Methods

### 2.1. Study Design, Setting, and Population

This multinational, web-based, and cross-sectional study was conducted to ascertain the hesitancy, conspiracy beliefs, and willingness to pay for the COVID-19 vaccines in six countries (Pakistan, India, Malaysia, Sudan, Egypt, and the Kingdom of Saudi Arabia) during the period of five months (April–August 2021). This study adheres Strengthening the Reporting of Observational Studies in Epidemiology(STROBE) checklist.

### 2.2. Inclusion and Exclusion Criteria

Any educated/literate (able to read or write in their national language) adult with access to social applications who agreed to fill in the e-questionnaire was eligible to participate. The exclusion criteria were (1) non-adults, (2) not being able to understand and complete the e-questionnaire, and (3) non-consenting individuals.

### 2.3. Sampling

An exponential, non-discriminate snowball sampling method was employed to recruit the study participants. This sampling method has been used previously in various cross-sectional studies [12,13,14]. The lead investigators of our research group belong to the countries included in this study, i.e., Pakistan: MS, ZUM, Malaysia: AAR, Sudan: MSM, India; DSP, Saudi Arabia: THM, YHK, and Egypt: AIMK. These investigators were assigned the responsibility of collecting the data from the respective countries. An orientation session was arranged with these investigators to familiarize them with the study design and data collection procedure. Each lead investigator constructed a data collection team consisting of members from our research group and followed the sampling process. Initially, each team selected a set of five individuals (first wave seeds), viz., broadly representing age, gender, and education level categories. The initial wave was selected from people personally or professionally known by the members of each research team. This set of invitees forwarded the questionnaire to five individuals (family members/relatives/neighbors/friends/colleagues) whom they considered suitable for this survey, and this second set disseminated the questionnaire in a similar manner, and so on.

### 2.4. Questionnaire Development, Translation, and Validation

#### 2.4.1. Questionnaire Development

The questionnaire was formulated by two investigators (MS and THM) through a comprehensive literature review [15,16,17,18,19]. The questionnaire was thoroughly reviewed by a panel of experts (one professor, one associate professor, and three assistant professors from the medical discipline) to assess the clarity, relevance, and comprehensibility of each section, and some details were improved. The data collection instrument (questionnaire) had the following sections (Supplementary File);

- Section-I: This section explained the aims, objectives, procedure, and nature of the study. All the study participants were informed that “*by completing this survey, you are agreeing to participate in this study*”.
- Section-II: This section consisted of eight items used to gather demographic details, e.g., age (18–25 years, 26–40 years, 41–60 years, and >60 years), gender (male/female/prefer not to say), level of education (primary, secondary, higher secondary, or graduated), residence (urban/suburban/rural), country of origin, presence of any chronic diseases (yes/no), and COVID-19 infection of oneself (yes/no) and/or family members (yes/no).
- Section-III: This section consisted seven items used to evaluate COVID-19-vaccine-related conspiracy beliefs (C19V-CBS) based on the published literature [20,21]. All the participants responded to these questions on a dichotomous scale (yes/no). Each incorrect response against these items was scored 1 or, otherwise, zero. The total conspiracy beliefs score ranged from 0 to 7, where a higher score indicated higher beliefs in conspiracy theories about the COVID-19 vaccines.
- Section-IV: This section contained one question (yes/no) used to determine the responder’s attitude towards governmental enforcement of vaccination for COVID-19.
- Section-V: This section had 10-item COVID-19 vaccine hesitancy scale (C19V-HS) that was developed from the published literature related to vaccine hesitancy [22,23]. A five-point Likert scale ranging from ‘1—strongly disagree’ to ‘5—strongly agree’ was used for each item. To calculate the C19V-HS score, we reverse-scored the first seven items (items 1–7: 5—strongly disagree, 4—disagree, 3—neutral, 2—agree, and 1-strongly agree) on the scale, so that higher scores indicated greater vaccine hesitancy on all items.
- Section-VI: This section consisted of three items (yes/no) used to determine the respondents’ willingness to pay for the COVID-19 vaccine.

#### 2.4.2. Translation and Validation

The English questionnaire was translated into the Urdu, Arabic, Hindi, and Bahasa Malay languages using forward-back translation methods by language experts. The internal consistency of the study tool was estimated by Cronbach’s alpha using the data of the first 50 responses from each language. The responses used for the validation of the study instrument were excluded from the final analysis. The results of our validation studies showed that all the translated versions of the study instruments had adequate internal consistency (Cronbach’s alpha > 0.7 for both the COVID-19 vaccine conspiracy belief scale (C19V-CBS) and C19V-HS).

### 2.5. Data Collection and Handling

The study instrument was shared with potential participants through various social media platforms (WhatsApp, Facebook Messenger, WeChat, etc.). The data collection instrument was available to participants for five months (April to August 2021). Each respondent was allowed to answer the survey only once, and no duplicates were included. After the data collection ended, responses from the online databases were downloaded on Microsoft Excel sheets. The results of each country were translated back into English and were pooled in one datasheet. Data from the Microsoft Excel sheets were coded and cleaned initially, and imported into the SPSS version 22 for analysis.

### 2.6. Data Analysis

The normality of the continuous data (C19V-CBS and C19V-HS) was determined by the Lilliefors-corrected Kolmogorov–Smirnov test and Shapiro–Wilk test. In addition, we also checked for skewness and kurtosis and performed a visual examination of the histograms. The continuous data were presented as the mean along with standard deviation (SD) or as median along with interquartile range (IQR) for normal (C19V-HS) and non-normal data (C19V-CBS), respectively. Categorical data were expressed in frequencies (*n*), along with the proportions (%).

For the inferential analysis, we either performed an independent *t*-test and one-way ANOVA or their non-parametric equivalent tests (Mann–Whitney U test and Kruskal–Wallis H test) for the continuous variables to compare two or more than two groups, respectively. Additionally, Tukey’s honest significant difference (HSD), the Games–Howell test, and Dunn’s test were used to determine significance between intergroup variables where applicable. The chi-square test was used to compare the categorical variables. We assessed the relationship between conspiracy beliefs and COVID-19 vaccine hesitancy using Spearman’s correlation. A two-sided *p*-value of less than 0.05 was considered significant for all the statistical tests.

### 2.7. Ethical Consideration

A permission to conduct this study was obtained from the Ethics Committee of the Faculty of Pharmacy, University of Lahore (REC/DPP/FOP/32). The data collection was performed anonymously. Personal information was not obtained, and all the study participants were ensured about the confidentiality of their data.

## 3. Results

### 3.1. Sample Characteristics

A total of 2481 complete responses were included in the analysis (Pakistan 24.1%, Saudi Arabia 19.5%, India 11.6%, Malaysia 8.1%, Sudan 19.3%, and Egypt 17.3%). The majority of the participants were ≤40 years old (18–25 years: 55.8%, 26–40 years: 28.5%). Moreover, there was a preponderance of females (57.1%) and participants with a tertiary level of education (92.8%). A total 337 (15.2%) participants reported being infected with COVID-19, whereas 36% reported having a family member or relative who had the infection (Table 1). The demographic characteristics stratified by the country of residence are shown in Supplementary Table S1.

### 3.2. COVID-19 Vaccine Related Conspiracy Beliefs

**Figure 1:**
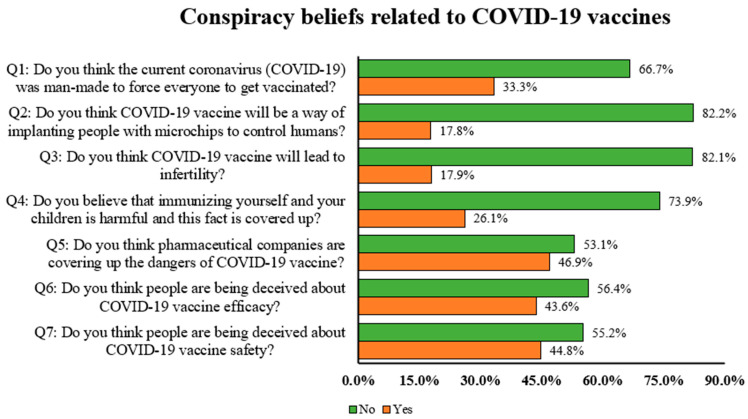
Participants’ responses with regard to COVID-19 vaccine conspiracy beliefs.

Figure 1 depicts the COVID-19-vaccine-related conspiracy beliefs among the study responders. One-third of the study population (33.3%) believed corona was a synthetic virus, 17.8% believed COVID-19 vaccines were a way of implanting people with microchips, 17.9% thought the vaccines would lead to infertility, 26.1% were of the opinion that immunizing themselves and their children was harmful and this fact was covered up, 46.9% were convinced that pharmaceutical companies were not being honest and hiding the dangers of COVID-19 vaccines, and 44–45% were seriously concerned about the COVID-19 vaccines’ safety and efficacy data. The COVID-19 vaccine beliefs stratified by country are provided in Supplementary Table S2.

**Figure 2:**
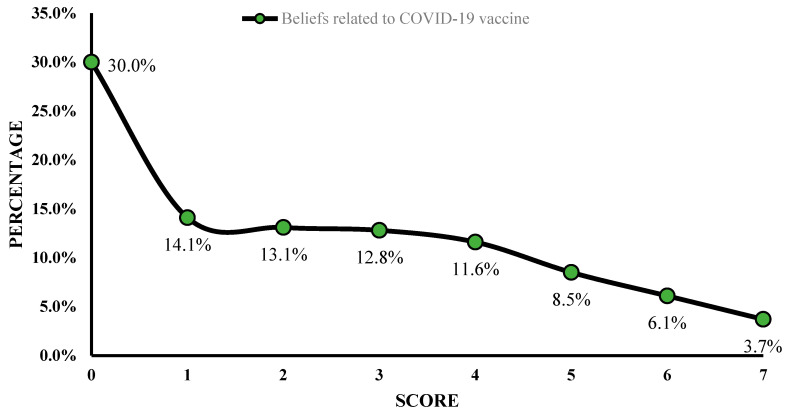
Distribution of vaccine conspiracy belief scores across the sample population.

The median C19V-CBS score was 2 (IQR 4, mean = 2.30 ± 2.12, range 0–7). The distribution of the conspiracy belief scores across the study population is shown in Figure 2. The ideal score (zero, indicating no conspiracy belief) was achieved among 30.0% of the population, whereas the highest score was observed in 3.7% of the study sample. As shown in Table 2, there was no significant difference in the C19V-CBS score according to the respondents’ gender and education level. Furthermore, the C19V-CBS score was not statistically different between the respondents who had or whose family members/relatives had contracted the infection during the pandemic and those who had not.

Malaysian respondents were found to have the lowest conspiracy belief score, followed by Saudi Arabia and India. In the multiple comparison (Supplementary Table S3), there was no significant difference (*p* > 0.05) in the vaccine conspiracy belief scores between respondents from Pakistan, India, Sudan, and Egypt. Malaysian and Saudi respondents had significantly less conspiracy beliefs than those from other countries. In the pairwise comparison using Dunn’s test, older age was found to be associated with less conspiracy beliefs (Supplementary Table S3). In addition, respondents belonging to rural areas were found to have greater beliefs in COVID-19 vaccine conspiracies than those from suburban (*p* = 0.001) or urban areas (*p* < 0.001).

### 3.3. COVID-19 Vaccine Hesitancy

**Figure 3:**
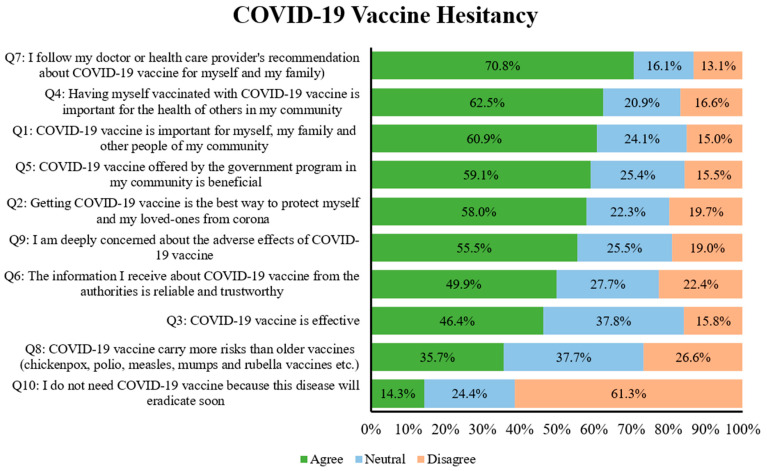
Participants’ responses related to COVID-19 vaccine hesitancy ranked according to the percentage of agreement.

As shown in Figure 3, 61% agreed that the COVID-19 vaccine was important for them, their family, and the health of all the people in their community, and 58% agreed that the vaccine was the best way to protect themselves and their loved ones from the virus. However, only 46% of the study participants considered COVID-19 vaccines to be totally effective against the infection. It was concerning to observe that only half of the study participants stated that the information they received about COVID-19 vaccines from their health authorities was reliable and trustworthy. However, it was encouraging to observe that the majority of the study participants agreed to follow their doctor’s recommendation to have the COVID-19 vaccine. Only 61% of the respondents disagreed (vaccine acceptors) with the statement that they do not need the COVID-19 vaccine, whereas 14.3% agreed (vaccine rejecters) and 24.4% chose neither agree nor disagree (fence-sitters/vaccine ambivalent).

**Figure 4:**
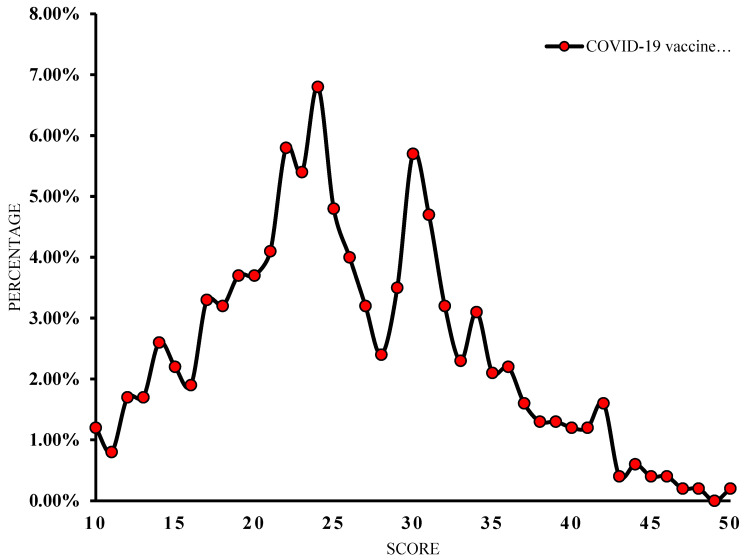
Distribution of COVID-19 vaccine hesitancy score across the study population.

The mean C19V-HS score was 25.93 ± 8.11 (range: 10–50). As shown in Figure 4, only 26% of the study participants achieved the ideal score (C19V-HS ≤ 20), indicating no vaccine hesitancy. The majority (45.7%) had C19V-HS scores of 21–30 and nearly 28% achieved a score greater than 30, indicating a higher degree of hesitancy.

Our comparison of the C19V-HS scores between the demographic variables is shown in Table 3. There was a significant difference in the vaccine hesitancy scores between countries and the COVID-19 infection of oneself and/or family member or relative variables. In the post hoc analysis, the vaccine hesitancy score was significantly higher among respondents from Egypt and Sudan than other countries (Supplementary Table S4).

**Figure 5:**
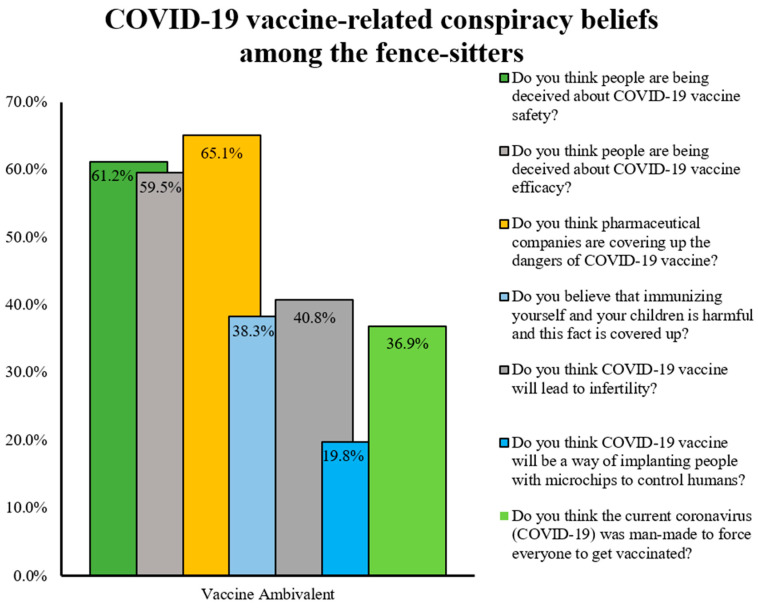
Most widespread COVID-19-vaccine-related conspiracy beliefs among the “fence-sitters”.

As expected, there was a significant positive correlation between conspiracy beliefs and vaccine hesitancy (Spearman’s rho = 0.547, *p* < 0.001). The vaccine hesitancy score was significantly higher among individuals who had conspiracy beliefs than those who did not (28.10 ± 7.64 vs. 20.86 ± 6.79, *p* < 0.001). Furthermore, conspiracy beliefs were significantly higher among vaccine rejecters and ambivalent individuals/fence-sitters than the vaccine acceptors (C19V-CBS mean rank: 1830.56 vs. 1506.13 vs. 997.94, respectively). The most widespread conspiracy beliefs among the vaccine ambivalent respondents are shown in Figure 5. The top three most common beliefs were mainly related to the dangers, safety, and efficacy of COVID-19 vaccines.

### 3.4. Government’s Enforcement of COVID-19 Vaccine

**Figure 6:**
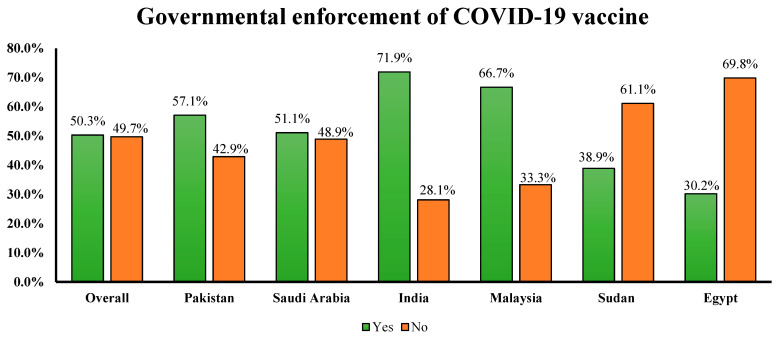
Respondents’ views on the government’s imposition of mandatory COVID-19 vaccination.

Almost half of the study participants (50.3%) were in favor of governmental enforcement of COVID-19 vaccines (Figure 6). Furthermore, respondents in favor of the government imposing mandatory COVID-19 vaccines had significantly lower scores on the vaccine conspiracy belief scale (*p* < 0.001) and C19V-HS scale (22.75 ± 7.24 vs. 29.13 ± 7.67; *p* < 0.001).

### 3.5. Willingness to Pay for COVID-19 Vaccine

**Figure 7:**
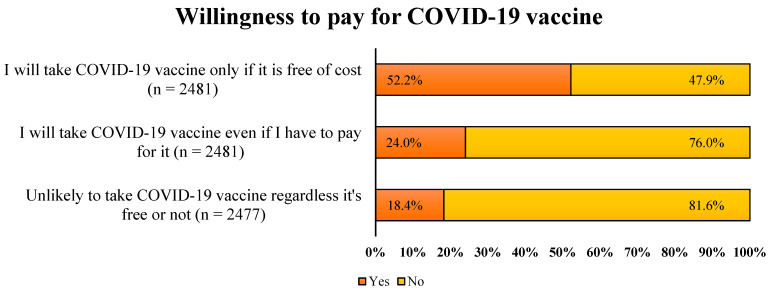
Respondents’ willingness to pay for the COVID-19 vaccine.

More than half (52.2%) of the study sample reported they would only take COVID-19 vaccine if it was free and only 24% were willing to pay for COVID-19 vaccines (Figure 7). Nearly 18% of the study participants stated that they may not take the vaccine regardless of whether it is free or not. The association of the respondents’ demographics with the willingness to pay for COVID-19 vaccines is shown in Table 4. A significantly higher percentage of respondents aged >40 years and those with a tertiary education were willing to pay for COVID-19 vaccine shots. Surprisingly, 18.7% of the respondents with a tertiary education refused to take the vaccine compared to 12.4% of those with a secondary education or less. Moreover, there was also a significantly lower frequency of males refusing COVID-19 vaccines than females (15.3 vs. 20.4, *p* = 0.001). The frequency with which respondents showed willingness to pay for the COVID-19 vaccine was highest for Malaysia followed by the KSA. The majority of the study participants who refused to get the vaccine were from Egypt (43.3%), followed by Sudan (28.3%), and Pakistan (12.8%). Interestingly, a significantly higher number of individuals who either were infected with SARS-CoV-2 or had a family member with the diseases during the pandemic refused to take the COVID-19 vaccine.

## 4. Discussion

Vaccine hesitancy (VH) has undermined efforts to immunize the population against COVID-19. The vaccine hesitancy has been identified by the World Health Organization (WHO) as one of the top 10 public health threats. that the VH may reverse the advancements made in controlling vaccine-preventable diseases (VPDs) [24]. This study was conducted during the time when various COVID-19 vaccines were readily available and accessible around the globe but, still, a considerable percentage of the population from the six selected countries have conspiracy beliefs and hesitancy regarding the COVID-19 vaccines. In this context, this study has pivotal implications for the healthcare authorities. We believe that addressing the conspiracy beliefs and vaccine hesitancy should be a continuous process regardless of the extent of vaccination coverage in the region. We suggest investigating vaccination readiness among the general population at regular intervals through the 7Cs model (confidence, complacency, constraints, calculation, collective responsibility, compliance, and conspiracy) [25]. Since vaccine hesitancy is not a fixed phenomenon, and anti-vax individuals may convert to pro-vax and vice versa, the estimation of vaccine acceptance should be a continuous process through longitudinal surveys.

The rapid spread of COVID-19-vaccine-related conspiracy theories may jeopardize the public health response due to, for example, unwillingness to be vaccinated against the virus. The COVID-19 pandemic resulted in emergence of various conspiracy theories related to SARS-CoV-2, as well as its vaccines. It has been well established that the proliferation of beliefs in conspiracies are linked with decreased social distancing motivations and increased vaccine rejection [26]. Our analysis showed that approximately 18% to 49% of the study population believed in one or more conspiracy theories. The covering up of the dangers of the vaccines by the pharmaceutical industries and deception about the safety and efficacy of the vaccines were the most common beliefs. On the other hand, implanting microchips and risks of infertility were the least common beliefs among the study participants. Only 30% of the study population did not believe in any conspiracy theory. Recent investigations have demonstrated that mistrust in governments, healthcare institutions, and the pharmaceutical industries remains a significant contributor of conspiracy beliefs and, hence, vaccine hesitancy [19,27,28,29].

The distribution and ranking of conspiracy beliefs correspond across all six countries that were included in our study, and these findings are coherent with the previous investigations from these regions [30,31,32,33,34]. Our analysis showed that young age, lower education, and rural residents had higher conspiracy belief scores. These results showed that demographic characteristics may relate to the magnitude of conspiracy beliefs. A national survey in Australia indicated a strong agreement between misinformation beliefs and participants of a young age, male gender, and lower education [35]. The factors associated with a high rate of conspiracy beliefs among these socio-demographic groups are debatable and require further investigation. However, it is imperative to understand the demographics of people who are more likely to believe and further disseminate misleading narratives or conspiracy theories [9]. This will aid the health authorities in providing evidence-based recommendations to take corrective measures to address the harms associated with misinformation or disinformation circulating about COVID-19 and its vaccines. Taken together, these findings indicate that conspiracy beliefs are circulating considerably in the countries included in this study, necessitating the need for curative measures to neutralize misleading narratives. On the other hand, it is very important to consider that our study relied on data from social media users, and people who use social media frequently may express more beliefs in conspiracy theories and misinformation [36]. There is a possibility that data from individuals who are not active on social media may generate variable findings. This relationship should be investigated in future studies.

Since vaccines are one of the key interventions used to reduce the severity of COVID-19, vaccine hesitancy is still a common phenomenon that varies by country and over time. Our analysis showed a preponderance of vaccine hesitancy in a substantial proportion of the study population, in which it was more prevalent in Sudan and Egypt. The higher magnitude of vaccine hesitancy in these two African countries is in alignment with previous investigations [37,38] and might be associated with the higher proportion of beliefs in conspiracy theories observed in our study. About 55% of the study population was deeply concerned about the side effects of COVID-19 vaccines and 36% believed that these vaccines carry more risks than older vaccines for other infectious diseases. A large body of literature has demonstrated that the fear of side effects related to the COVID-19 vaccines is a significant contributor to vaccine hesitancy [39,40]. This concern can be addressed effectively through behaviorally informed messages. For example, a simple message such as “All coronavirus vaccines available in the country are proven to be safe” through media and cellular services may prove to be an effective measure. Moreover, sharing real-world pharmacovigilance data would also boost the confidence of the public in the safety of the COVID-19 vaccines. It is important to note that half of the study population had doubt about the reliability and trustworthiness of COVID-19-vaccine-related information disseminated by the health authorities. This finding might be related to the reason for which most of the respondents in this study reported being deceived about the safety and efficacy of COVID-19 vaccines. In this context, restoring the trust of the general population in healthcare authorities is highly recommended, as this could be translated into trust in vaccines. Ensuring the transparency and integrity of advisory bodies for the COVID-19 pandemic and community participation in these bodies would aid in efforts to escalate the trust in authorities, as well as vaccines.

Interestingly, about 71% of the study population reported following the recommendations of their healthcare providers to be vaccinated against COVID-19. A recent analysis based on a telephonic survey of 340,543 Americans demonstrated that clinicians’ recommendations increase the COVID-19 vaccination rate [41]. Moreover, the clinician’s encouragement can also help to boost the confidence of the general community in the COVID-19 vaccines [42]. In this context, considering the perception of healthcare providers towards vaccine hesitancy is of paramount importance. Recent investigations have indicated a high rate of COVID-19 vaccine hesitancy among healthcare professionals. The concerns on the safety, efficacy, and rapid pace of vaccine development were found to be major contributors to vaccine hesitancy among healthcare professionals [43,44]. The health authorities should primarily focus on alleviating any concerns and doubts regarding COVID-19 vaccines among healthcare professionals before encouraging them to recommend vaccination to the public.

The vaccine ambivalent attitude gained substantial popularity during the COVID-19 pandemic and refers to individuals who are neither vaccine acceptors nor rejecters but fall on the borderline of vaccine hesitancy [45]. This population can shift to vaccine rejecter or acceptor status based on the circumstances. Our analysis showed that about 16% to 38% of the study population was neutral on the vaccine hesitancy scales, thereby meeting the criteria for vaccine ambivalence. It must be noted that the state of vaccine ambivalence should not be considered irrational or anti-vax. The phenomenon of vaccine ambivalence often reflects legitimate doubts and concerns related to the vaccines. In this context, it should be considered as an opportunity to implement the educational campaigns to clarify these doubts and concerns [46]. Some studies found a higher proportion of vaccine ambivalent individuals than rejecters, indicating that they would be a more responsive target for strategies aiming to increase vaccine uptake among the population [45]. Addressing the vaccine ambivalence among people may be an efficient way of increasing vaccine coverage. Moreover, vaccine ambivalence will be an important target in the identification of strategies to boost vaccine uptake [47], particularly in the place where the vaccination booster program is planned or rolled out. Our study emphasizes that countering the vaccine ambivalence phenomenon must be considered a priority measure during the design and implementation of immunization policies against COVID-19.

It is pertinent to mention that half of the study population was against the vaccine mandate and reported that it is not acceptable to force individuals to vaccinate. Government-mandated proof-of-vaccination requirements or certificates are linked with increased vaccine uptake [48]. We believe that vaccine mandates are the need of the hour and can counter vaccine hesitancy in various regions across the globe. Previously, a survey of 19 countries (55% of the world’s population) showed considerable vaccine hesitancy when the respondents were asked “If a COVID-19 vaccine is proven safe and effective and is available, will you take it?” However, it was observed that vaccine uptake increased the threshold of hesitancy in these countries following the vaccine mandates [49]. Our study showed that Indians, Malaysians, Pakistanis, and Saudis were in favor of vaccine mandates. However, the vaccine mandate was considered unacceptable by most of the population from Sudan (61.1%) and Egypt (69.8%). It can be hypothesized that vaccine hesitancy, conspiracy beliefs, and the rejection of the vaccine mandates may interlink with each other. Our analysis showed the highest level of vaccine hesitancy in Sudan and Egypt as compared to the other four countries. A recent investigation from Sudan demonstrated the unsatisfactory vaccination rate in the country, which may primarily be attributed to the population’s concerns about the safety of vaccines [50]. Another study calculated a high level of COVID-19 vaccine hesitancy among medical students in Sudan [38]. In addition, the distrust in the government due to the recent political crisis might be another reason for the refusal of the vaccine mandate in Sudan [51]. It is worth mentioning that vaccinations are not mandatory in Sudan thus far, and health authorities may face some challenges when imposing vaccine mandates in the region, for which proactive measures are deemed necessary. Likewise, a meta-analysis of six studies from Egypt indicated a low rate of COVID-19 vaccine acceptance, with a pooled acceptability proportion of 42.6% [37]. This high magnitude of vaccine hesitancy might be a potential perpetrator contributing to the lack of acceptance of the vaccine mandates. The same meta-analysis reported a high rate of acceptance of the COVID-19 vaccine in India (76.7%), and it may be a possible reason for the finding that most of the population from India (71.9%) in our study agreed to the government-imposed vaccine mandate.

Willingness to pay (WTP) for COVID-19 vaccines is of paramount importance in ensuring the vaccines’ acceptance and valuing. Our analysis showed that half of the study population agreed to take the COVID-19 vaccines only if they are free, and only one-quarter of the study population agreed to pay for the vaccines. These findings demonstrate that most of the study population did not want to pay for the vaccines. It is essential to understand the WTP for COVID-19 vaccines, as it is correlated with the vaccines’ acceptance. The low WTP in our study might be associated with several factors, specifically vaccine hesitancy and conspiracy beliefs. In addition, the low levels of WTP might be attributed to the fact that most of the study population belonged to low-income countries (Pakistan, India, Sudan, Egypt). This hypothesis can be confirmed by the evidence showing that the WTP was highest in Malaysia and Saudi Arabia. The economic status of the individual is another factor, but a recent review from lower-middle, upper-middle- and high-income countries showed that the GDP per capita of the country is not the sole factor driving the WTP [52]. It must be noted that we did not estimate the extent of the WTP; however, the previous investigations showed a low WTP, ranging from USD 3.12 to 7 in Pakistan, USD 6.81 in India, and USD 30 in Malaysia, as compared to USD 332 in Chile and USD 318 in America [8,53,54,55]. We believe that the COVID-19 vaccines should be available free of cost, and any consideration of payment should be delayed until the declaration of the end of pandemic. The vaccine hesitancy phenomenon may be more profound if people have to pay to get vaccinated [54]. In this context, the pricing policies on the COVID-19 vaccines should be designed cautiously, taking into consideration the economic status, vaccine hesitancy pattern, and prevalence of conspiracy beliefs in the region.

It is important to note that conspiracy beliefs may directly relate to vaccine hesitancy or rejection. Our analysis showed a positive correlation between conspiracy belief scores and vaccine hesitancy scores (r = 0.547). However, this hypothesis should be tested by taking into account various covariates during the analysis.

The findings of this study should be interpreted in light of a few shortcomings. The results rely on the participants’ self-reports with regard to their vaccination intentions, which may fluctuate, with the passage of time, with respect to their actual vaccination behavior. The sample cannot be claimed to be fully representative due to the non-probability sampling technique used in this study. There is a possibility that the initial waves or seeds of the sample recruited people with similar traits; therefore, the propensity for selection bias and the margin of error cannot be disregarded when interpreting the results. In addition, the dynamic and heterogeneous progress of vaccination campaigns across the countries included in this study may have caused disparities in the composition and representativeness in each target country, thereby precluding the generalizability of the findings. The timing of the data collection should also be considered an important factor interfering with the perception of the participants. For example, Sudan was experiencing a political crisis at the time of the data collection, and the responses may vary if they were collected under normal circumstances. Likewise, the number of cases in the targeted countries may also impact the perceived need for getting vaccinated. The generalizability of the findings is also affected by the lower response from Malaysia and India. We relied on only seven common rumors related to COVID-19 to estimate the beliefs in conspiracy theories. Extending the list of rumors according to the region may result in variable belief scores. The education level among most of the participants was at the tertiary level, and the findings may not represent data from individuals with lower education levels. Similarly, most of the respondents were young and urban residents; therefore, our findings may not relate to the elderly and rural populations of the countries. Since this study recruited participants through web-based survey, there is a propensity that people having less interaction with social media are not well represented in our data. Nevertheless, this study offers new insights into our understanding of conspiracy beliefs, vaccine hesitancy, perceptions regarding vaccine mandates, and willingness to pay in six countries across Asia and Africa. The findings of our study provide pivotal suggestions for future research and public health interventions. These directives must be considered by the health authorities of these 6 countries, which account for approximately 20% of the world’s population. In addition, our findings can be utilized as an initial motivation and guide during the design and implementation of future studies and vaccine rate improvement campaigns. The limitations related to this study should be addressed in future studies. Last but not least, the large sample size in this study ensures the power of the inferential analysis. Likewise, the consistency of the results from different countries that are geographically close and socioeconomically similar confirm the reliability of our survey.

## 5. Conclusions

Our results underscore the high prevalence of conspiracy beliefs and vaccine hesitancy in the targeted countries. The covering up of the dangers of vaccines by the pharmaceutical industries and deception about the safety and efficacy of the vaccines were the most common conspiracy beliefs. However, the implanting of microchips and the risks of infertility were the least common beliefs. Vaccine hesitancy was more prevalent in Sudan and Egypt. Approximately half of the study population were against the vaccine mandate, particularly residents of Sudan and Egypt. Only 24% of the study population showed a willingness to pay (WTP) for the COVID-19 vaccines, whereas residents from Malaysia and Saudi Arabia indicated a higher WTP. Given the fact that vaccines are the key interventions used to reduce the severity of COVID-19, higher hesitancy and conspiracy beliefs may interfere with the ongoing immunization campaigns. Our findings demonstrate a dire need for aggressive measures to counter the conspiracy beliefs and factors underlying vaccine hesitancy in the community. There is a need for structured public education programs, positive peer influence, the restoration of the trust of the public in health authorities, and ascertainment of the magnitude of public perceptions towards COVID-19 vaccine campaigns. Considering the dynamic nature of the pandemic and the interplay between ever-changing factors that affect vaccine acceptance, our findings should be replicated in future studies to measure the trajectory of vaccine hesitancy over time.

## Figures and Tables

**Table 1:** Demographic details of the study participants.

Variables	*n* (%)
Age (years)	
≤25	1385 (55.8)
26–40	706 (28.5)
41–60	357 (14.4)
>60	33 (1.3)
Gender	
Male	1065 (42.9)
Female	1416 (57.1)
Education	
Higher secondary or less	178 (7.2)
Tertiary	2303 (92.8)
Country of residence	
Pakistan	599 (24.1)
Saudi Arabia	485 (19.5)
India	288 (11.6)
Malaysia	201 (8.1)
Sudan	478 (19.3)
Egypt	430 (17.3)
Area/locality	
Rural	398 (16.0)
Suburban	342 (13.8)
Urban	1741 (70.2)
Got infected with COVID-19	
Yes	377 (15.2)
No	2104 (84.8)
Family member infected with COVID-19	
Yes	892 (36.0)
No	1589 (64.0)

**Table 2:** Respondents’ beliefs about the COVID-19 vaccine.

Variables	Median (IQR)	Mean Rank *	*p*-Value
Age (years)			0.004 **
≤25	2 (4)	1272.44	
26–40	2 (4)	1235.99	
>40	1 (3)	1138.41	
Gender			0.111 ^¥^
Male	2 (4)	1266.91	
Female	2 (4)	1221.51	
Education			0.050 ^¥^
Higher secondary or less	2 (3)	1340.40	
Tertiary	2 (4)	1233.32	
Country of residence			<0.001 **
Pakistan	2 (3)	1328.17	
Saudi Arabia	1 (3)	1053.95	
India	2 (3)	1279.71	
Malaysia	1 (2)	959.51	
Sudan	2 (4)	1319.31	
Egypt	3 (4)	1349.15	
Area/locality			<0.001 **
Rural	3 (3)	1382.68	
Suburban	2 (4)	1212.14	
Urban	2 (4)	1214.28	
Got infected with COVID-19			0.190 ^¥^
Yes	2 (4)	1284.69	
No	2 (4)	1233.17	
Family member infected with COVID-19			0.839 ^¥^
Yes	2 (4)	1237.18	
No	2 (4)	1243.14	

IQR: interquartile range; * higher mean rank indicates greater beliefs in the COVID-19-vaccine-related conspiracies; ^¥^ Mann–Whitney U test; ** Kruskal–Wallis H test.

**Table 3:** Comparison of COVID-19 vaccine hesitancy scores between demographics.

Variables	Mean ± SD	*p*-Value
Age (years)		0.122 *
≤25	26.03 ± 7.64	
26–40	26.19 ± 8.26	
>40	25.07 ± 9.34	
Gender		0.166
Male	25.66 ± 8.41	
Female	26.12 ± 7.88	
Education		0.153
Higher secondary or less	25.09 ± 7.97	
Tertiary	25.99 ± 8.12	
Country of residence		<0.001 *
Pakistan	24.85 ± 6.89	
Saudi Arabia	23.73 ± 8.85	
India	23.39 ± 6.75	
Malaysia	21.28 ± 6.25	
Sudan	28.32 ± 8.63	
Egypt	31.11 ± 6.27	
Area/locality		0.797 *
Rural	25.91 ± 7.64	
Suburban	25.68 ± 7.40	
Urban	25.98 ± 8.35	
Got infected with COVID-19		0.001
Yes	27.21 ± 8.33	
No	25.70 ± 8.05	
Family member infected with COVID-19		0.002
Yes	26.59 ± 8.10	
No	25.56 ± 8.09	

* Welch’s ANOVA was used instead of classic ANOVA, as the assumption of homoscedasticity was violated.

**Table 4:** Association of the respondents’ demographics with the willingness to pay for COVID-19 vaccines.

Variables	Willingness to Pay for COVID-19 Vaccine								
	Getting Vaccinated Only If It Is Free, *n* (%)		*p*-Value	Willing to Pay for the Vaccine, *n* (%)		*p*-Value	Unlikely to Take Vaccine, *n* (%)		*p*-Value
	Yes	No		Yes	No		Yes	No	
Age (years)			<0.001			<0.001			0.675
≤25	770 (55.6)	615 (44.4)		285 (20.6)	1100 (79.4)		259 (18.7)	1124 (81.3)	
26–40	351 (49.7)	355 (50.3)		184 (26.1)	522 (73.9)		121 (17.2)	584 (82.8)	
>40	173 (44.4)	217 (55.6)		126 (32.3)	264 (67.7)		72 (18.5)	317 (81.5)	
Gender			0.057 *			0.887 *			0.001 *
Male	579 (54.4)	486 (45.6)		257 (24.1)	808 (75.9)		163 (15.3)	900 (84.7)	
Female	715 (50.5)	701 (49.5)		338 (23.9)	1078 (76.1)		289 (20.4)	1125 (79.6)	
Education			0.003			0.036 *			0.034 *
Higher secondary or less	112 (62.9)	66 (37.1)		31 (17.4)	147 (82.6)		22 (12.4)	156 (87.6)	
Tertiary	1182 (51.3)	1121 (48.7)		564 (24.5)	1739 (75.5)		430 (18.7)	1869 (81.3)	
Country of residence			<0.001			<0.001			<0.001
Pakistan	353 (58.9)	246 (41.1)		145 (24.2)	454 (75.8)		76 (12.8)	520 (87.2)	
Saudi Arabia	295 (60.8)	190 (39.2)		137 (28.2)	348 (71.8)		32 (6.6)	453 (93.4)	
India	197 (68.4)	91 (31.6)		72 (25.0)	216 (75.0)		10 (3.5)	278 (96.5)	
Malaysia	115 (57.2)	86 (42.8)		71 (35.3)	130 (64.7)		13 (6.5)	188 (93.5)	
Sudan	181 (37.9)	297 (62.1)		122 (25.5)	356 (74.5)		135 (28.3)	342 (71.7)	
Egypt	153 (35.6)	277 (64.4)		48 (11.2)	382 (88.8)		186 (43.3)	244 (56.7)	
Area/locality			<0.001			<0.001			0.135
Rural	238 (59.8)	160 (40.2)		68 (17.1)	330 (82.9)		70 (17.6)	328 (82.4)	
Suburban	213 (62.3)	129 (37.7)		68 (19.9)	274 (80.1)		50 (14.6)	292 (85.4)	
Urban	843 (48.4)	898 (51.6)		459 (26.4)	1282 (73.6)		332 (19.1)	1405 (80.5)	
Was infected with COVID-19			0.004 *			0.556*			<0.001 *
Yes	171 (45.4)	206 (54.6)		95 (25.5)	282 (74.8)		98 (26.0)	279 (74.0)	
No	1123 (53.4)	982 (46.6)		500 (23.8)	1604 (76.2)		354 (16.9)	1746 (83.1)	
Family member was infected with COVID-19			0.003 *			0.732			<0.001 *
Yes	430 (48.2)	462 (51.8)		210 (23.5)	682 (76.5)		196 (22.0)	695 (78.0)	
No	864 (54.4)	725 (45.6)		385 (24.2)	1204 (75.8)		256 (16.1)	1330 (83.9)	

* Fisher’s exact test.

## Data Availability

Not applicable.

## Supplementary Materials

The following supporting information can be downloaded at: https://www.mdpi.com/article/10.3390/vaccines10111866/s1, Table S1: Demographic characteristics stratified by country of residence; Table S2: COVID-19 vaccine conspiracy beliefs stratified by countries of residence; Table S3: Pairwise comparisons of COVID-19 vaccine conspiracy belief score using Dunn’s test; Table S4: Multiple comparisons of vaccine hesitancy scores using the Games–Howell test.
