# Supplementary material for: Evaluation of Conspiracy Beliefs, Vaccine Hesitancy, and Willingness to Pay towards COVID-19 Vaccines in Six Countries from Asian and African Regions: A Large Multinational Analysis
Source: Vaccines (Basel). 2022 Nov 4;10(11):1866. doi: 10.3390/vaccines10111866 (PMC9694922; doi:10.3390/vaccines10111866)
Supplement: Supplementary file 1 [file vaccines-10-01866-s001.zip › vaccines-1912028-supplementary.pdf]

vaccines

MDPI

Supplementary Tables

Table S1. Demographic characteristics stratified by country of residence.

Variables	Pakistan (*n* = 599)	Saudi Arabia (*n* = 485)	India (*n* = 288)	Malaysia (*n* = 201)	Sudan (*n* = 478)	Egypt (*n* = 430)	*p*-Value
**Age (years)**							
⩽25	427 (42.3)	205 (42.3)	200 (69.4)	139 (69.2)	64 (13.4)	350 (81.4)	**<0.001**
26–40	140 (23.4)	172 (35.5)	63 (21.9)	38 (18.9)	224 (46.9)	69 (16.0)	
>40	32 (5.3)	108 (22.3)	25 (8.7)	24 (39.7)	190 (39.7)	11 (2.6)	
**Gender**							
Male	229 (38.2)	181 (37.3)	201 (69.8)	62 (30.8)	250 (52.3)	142 (33.0)	**<0.001**
Female	370 (61.8)	304 (62.7)	87 (30.2)	139 (69.2)	228 (47.7)	288 (67.0)	
**Education**							
Higher secondary or less	34 (5.7)	55 (11.3)	46 (16.0)	10 (5.0)	22 (4.6)	11 (2.6)	**<0.001**
Tertiary	565 (94.3)	430 (88.7)	242 (84.0)	191 (95.0)	456 (95.4)	419 (97.4)	
**Residence**							
Rural	105 (17.5)	22 (4.5)	130 (45.1)	11 (5.5)	12 (2.5)	118 (27.4)	**<0.001**
Suburban	56 (9.3)	72 (14.8)	57 (19.8)	47 (23.4)	32 (6.7)	78 (18.1)	
Urban	438 (73.1)	391 (80.6)	101 (35.1)	143 (71.1)	434 (90.8)	234 (54.4)	
**Was infected with COVID-19**							
Yes	85 (14.2)	78 (16.1)	7 (2.4)	9 (4.5)	112 (23.4)	86 (20.0)	**<0.001**
No	514 (85.8)	407 (83.9)	281 (97.6)	192 (95.5)	366 (76.6)	344 (80.0)	
**Any Family member or relative infected with COVID-19**							**<0.001**
Yes	211 (35.2)	237 (48.9)	22 (7.6)	28 (13.9)	186 (38.9)	208 (48.4)	
No	388 (64.8)	248 (51.1)	266 (92.4)	173 (86.1)	292 (61.1)	222 (51.6)	

Table S2. COVID-19 vaccine conspiracy beliefs stratified by countries of residence.

Questions	Answer *n* (%)	Country of Residence						
		Pakistan	Saudi Arabia	India	Malaysia	Sudan	Egypt	*p*-Value
Do you think COVID-19 was manmade to force everyone to get vaccinated?	Yes	204 (34.1)	173 (35.7)	178 (61.8)	62 (30.8)	172 (36.0)	104 (24.2)	**<0.001**
	No	395 (65.9)	312 (64.3)	110 (38.2)	139 (69.2)	306 (64.0)	326 (75.8)	
Do you think the COVID-19 vaccine is a way of implanting people with microchips to control humans?	Yes	136 (22.7)	61 (12.6)	61 (21.2)	18 (9.0)	100 (20.9)	66 (15.3)	**<0.001**
	No	463 (77.3)	424 (87.4)	227 (78.8)	183 (91.0)	378 (79.1)	364 (84.7)	
Do you think the COVID-19 vaccine will lead to infertility?	Yes	128 (21.4)	90 (18.6)	49 (17.0)	13 (6.5)	88 (18.4)	76 (17.7)	**<0.001**
	No	471 (78.6)	395 (81.4)	239 (83.0)	188 (93.5)	390 (81.6)	354 (82.3)	
Do you believe that immunizing yourself and your children is harmful and this fact is covered up?	Yes	150 (25.0)	97 (20.0)	63 (21.9)	23 (11.4)	170 (35.6)	144 (33.5)	**<0.001**
	No	449 (75.0)	388 (80.0)	225 (78.1)	178 (88.6)	308 (64.4)	286 (66.5)	
Do you think pharmaceutical companies are covering up the dangers of COVID-19 vaccines?	Yes	308 (51.4)	191 (39.4)	102 (35.4)	54 (26.9)	248 (51.9)	260 (60.5)	**<0.001**
	No	291 (48.6)	294 (60.6)	186 (64.6)	147 (73.1)	230 (48.1)	170 (39.5)	
Do you think people are being deceived about the COVID-19 vaccines' efficacy?	Yes	296 (49.4)	129 (26.6)	141 (49.0)	60 (29.9)	218 (45.6)	238 (55.3)	**<0.001**
	No	303 (50.6)	356 (73.4)	147 (51.0)	141 (70.1)	260 (54.4)	192 (44.7)	
Do you think people are being deceived about the COVID-19 vaccines' safety?	Yes	303 (50.6)	135 (27.8)	147 (51.0)	64 (31.8)	224 (46.9)	238 (55.3)	**<0.001**
	No	296 (49.4)	350 (72.2)	141 (49.0)	137 (68.2)	254 (53.1)	192 (44.7)	

Table S3. Pairwise comparisons of COVID-19 vaccine conspiracy belief score using Dunn's test

Pairwise comparisons	Mean Rank	P-value
	Age (years)	
< 25 vs 26-40	1272.44 vs 1235.99	0.262
< 25 vs > 40	1272.44 vs 1138.41	0.001
26-40 vs > 40	1235.99 vs 1138.41	0.028
	Country of residence	
Pakistan vs Saudi Arabia	1328.17 vs 1053.95	< 0.001
Pakistan vs India	1328.17 vs 1279.71	0.336
Pakistan vs Malaysia	1328.17 vs 959.51	< 0.001
Pakistan vs Sudan	1328.17 vs 1319.31	0.837
Pakistan vs Egypt	1328.17 vs 1349.15	0.637
Saudi Arabia vs India	1053.95 vs 1279.71	< 0.001
Saudi Arabia vs Malaysia	1053.95 vs 959.51	0.109
Saudi Arabia vs Sudan	1053.95 vs 1319.31	< 0.001
Saudi Arabia vs Egypt	1053.95 vs 1349.15	< 0.001
India vs Malaysia	1279.71 vs 959.51	< 0.001
India vs Sudan	1279.71 vs 1319.31	0.450
India vs Egypt	1279.71 vs 1349.15	0.195
Malaysia vs Sudan	959.51 vs 1319.31	< 0.001
Malaysia vs Egypt	959.51 vs 1349.15	< 0.001
Sudan vs Egypt	1319.31 vs 1349.15	0.523
	**Area/locality**	
Rural vs Suburban	1382.68 vs 1212.14	0.001
Rural vs Urban	1382.68 vs 1214.28	< 0.001
Suburban vs Urban	1212.14 vs 1214.28	0.958

Table S4. Multiple comparisons of vaccine hesitancy scores using the Games–Howell test.

I	J	Mean Difference (I-J)	Std. Error	*p*-Value	95% Confidence Interval	
					Lower Bound	Upper Bound
		Country of Residence				
Pakistan	Saudi Arabia	1.12152	0.49058	0.201	−0.2795	2.5226
	India	1.46600 *	0.48743	0.033	0.0723	2.8597
	Malaysia	3.57281 *	0.52298	0.000	2.0747	5.0709
	Sudan	−3.47494 *	0.48475	0.000	−4.8593	−2.0905
	Egypt	−6.26021 *	0.41309	0.000	−7.4398	−5.0807
Saudi Arabia	Pakistan	−1.12152	0.49058	0.201	−2.5226	0.2795
	India	0.34448	0.56552	0.990	−1.2714	1.9604
	Malaysia	2.45129 *	0.59643	0.001	0.7453	4.1573
	Sudan	−4.59646 *	0.56321	0.000	−6.2047	−2.9882
	Egypt	−7.38173 *	0.50286	0.000	−8.8179	−5.9455
India	Pakistan	−1.46600 *	0.48743	0.033	−2.8597	−0.0723
	Saudi Arabia	−0.34448	0.56552	0.990	−1.9604	1.2714
	Malaysia	2.10681 *	0.59385	0.006	0.4072	3.8064

	Sudan	−4.94094 *	0.56048	0.000	−6.5425	−3.3394
	Egypt	−7.72621 *	0.49979	0.000	−9.1552	−6.2972
Malaysia	Pakistan	−3.57281 *	0.52298	0.000	−5.0709	−2.0747
	Saudi Arabia	−2.45129 *	0.59643	0.001	−4.1573	−0.7453
	India	−2.10681 *	0.59385	0.006	−3.8064	−0.4072
	Sudan	−7.04775 *	0.59165	0.000	−8.7402	−5.3553
	Egypt	−9.83302 *	0.53452	0.000	−11.3638	−8.3022
Sudan	Pakistan	3.47494 *	0.48475	0.000	2.0905	4.8593
	Saudi Arabia	4.59646 *	0.56321	0.000	2.9882	6.2047
	India	4.94094 *	0.56048	0.000	3.3394	6.5425
	Malaysia	7.04775 *	0.59165	0.000	5.3553	8.7402
	Egypt	−2.78527 *	0.49718	0.000	−4.2053	−1.3653
Egypt	Pakistan	6.26021 *	0.41309	0.000	5.0807	7.4398
	Saudi Arabia	7.38173 *	0.50286	0.000	5.9455	8.8179
	India	7.72621 *	0.49979	0.000	6.2972	9.1552
	Malaysia	9.83302 *	0.53452	0.000	8.3022	11.3638
	Sudan	2.78527 *	0.49718	0.000	1.3653	4.2053

* The mean difference is significant at the 0.05 level.

vaccines

MDPI

Data Collection Instrument

Section I:

The aim of this study is to assess the attitudes towards the COVID-19 vaccines among the general public. The participation in the study is completely voluntary. The study is purely for research purpose and all information provided will be treated with utmost confidentiality.

Please note

- You must be 18 or older to participate in this study.
- By completing this survey, you are consenting to participate in this study.

Section II:

Age (Years): (A) 18-25 (B) 26-40 (C) 41-60 (D) > 60

Gender: (A) Male (B) Female (C) Prefer not to say

Education level: (A) Primary (B) Secondary or higher secondary (C) Tertiary

Residence: (A) Urban (B) Sub-urban (C) Rural

Country of residence: ________________________________

Are you suffering from any chronic diseases (hypertension, diabetes, heart disease, respiratory disease etc.)?

(A) Yes (B) No

Did you get infected with COVID-19?

(A) Yes (B) No

Did anyone in your family get infected with COVID-19?

(A) Yes (B) No

Section III:

Do you think the current coronavirus (COVID-19) was man-made to force everyone to get vaccinated?

(A) Yes (B) No

Do you think COVID-19 vaccine will be a way of implanting people with microchips to control humans?

(A) Yes (B) No

Do you think COVID-19 vaccine will lead to infertility?

(A) Yes (B) No

Do you believe that immunizing yourself and your children is harmful and this fact is covered up?

(A) Yes (B) No

Do you think pharmaceutical companies are covering up the dangers of COVID-19 vaccine?

(A) Yes (B) No

Do you think people are being deceived about COVID-19 vaccine efficacy?

(A) Yes (B) No

Do you think people are being deceived about COVID-19 vaccine safety?

(A) Yes (B) No

Section IV:

Do you think it is acceptable for the government to force everyone to get COVID-19 vaccine?

(A) Yes (B) No

Section V:

COVID-19 vaccine is important for myself, my family and other people of my community.

(A) Strongly disagree (B) Disagree (C) Neutral (D) Agree (E) Strongly agree

Getting COVID-19 vaccine is the best way to protect myself and my loved-ones from corona.

(A) Strongly disagree (B) Disagree (C) Neutral (D) Agree (E) Strongly agree

COVID-19 vaccine is effective.

(A) Strongly disagree (B) Disagree (C) Neutral (D) Agree (E) Strongly agree

Having myself vaccinated with COVID-19 vaccine is important for the health of others in my community.

(A) Strongly disagree (B) Disagree (C) Neutral (D) Agree (E) Strongly agree

COVID-19 vaccine offered by the government program in my community is beneficial.

(A) Strongly disagree (B) Disagree (C) Neutral (D) Agree (E) Strongly agree

The information I receive about COVID-19 vaccine from the authorities is reliable and trustworthy.

(A) Strongly disagree (B) Disagree (C) Neutral (D) Agree (E) Strongly agree

I will follow my doctor or health care provider's recommendation about COVID-19 vaccine for myself and my family.

(A) Strongly disagree (B) Disagree (C) Neutral (D) Agree (E) Strongly agree

COVID-19 vaccine carry more risks than older vaccines (chickenpox, polio, measles, mumps and rubella vaccines etc.)

(A) Strongly disagree (B) Disagree (C) Neutral (D) Agree (E) Strongly agree

I am deeply concerned about serious adverse effects of COVID-19 vaccine.

(A) Strongly disagree (B) Disagree (C) Neutral (D) Agree (E) Strongly agree

I do not need COVID-19 vaccine because this disease will eradicate soon

(A) Strongly disagree (B) Disagree (C) Neutral (D) Agree (E) Strongly agree

Section VI:

I am willing to take COVID-19 vaccine only if it will be free of cost.

(A) Yes (B) No

I am willing to take COVID-19 vaccine even if I will have to pay for it.

(A) Yes (B) No

I will not take COVID-19 vaccine whether it’s free or not.

(A) Yes (B) No

Thank you for participating in this survey.

Authors Notes: This data collection form can be used for education and research purposes with appropriate citations.
